# Supplementary material for: ANKZF1 helps to eliminate stress-damaged mitochondria by LC3-mediated mitophagy
Source: Cell Death Discov. 2025 Jul 29;11:349. doi: 10.1038/s41420-025-02638-y (PMC12307761; doi:10.1038/s41420-025-02638-y)
Supplement: Supplementary file 1 — Supplementary Information [file 41420_2025_2638_MOESM1_ESM.pdf]

Supplementary Materials

ANKZF1 helps to eliminate stress-damaged mitochondria by LC3-mediated mitophagy

Mudassar Ali[1], Anjali[1] and Koyeli Mapa[1*]

1. Protein Homeostasis Laboratory, Department of Life Sciences, School of Natural Sciences, Shiv Nadar Institution of Eminence, Delhi-NCR, Greater Noida, Gautam Buddha Nagar, Uttar Pradesh 201314, India.

* Correspondence: koyeli.mapa@snu.edu.in

Running title:

ANKZF1 interacts with LC3 to clear damaged mitochondria

Figure S1

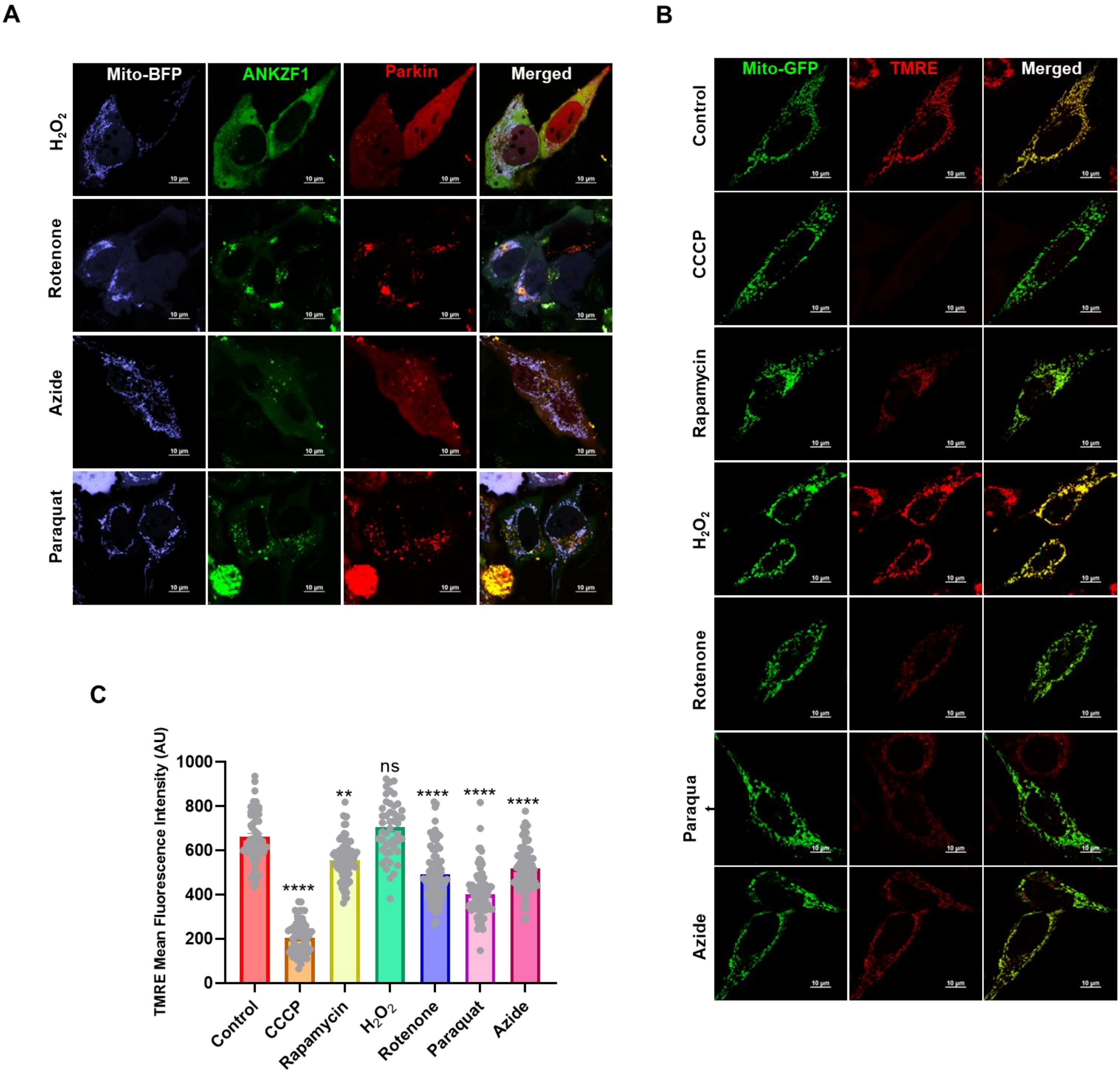

A
Mito-BFP
ANKZF1
Parkin
Merged
H2O2
Rotenone
Azide
Paraquat
10 μm
B
Mito-GFP
TMRE
Merged
Control
CCCP
Rapamycin
H2O2
Rotenone
Paraqua t
Azide
C
TMRE Mean Fluorescence Intensity (AU)
1000
800
600
400
200
0
****
**
ns
****
****
****
Control
CCCP
Rapamycin
H2O2
Rotenone
Paraquat
Azide

Figure S2

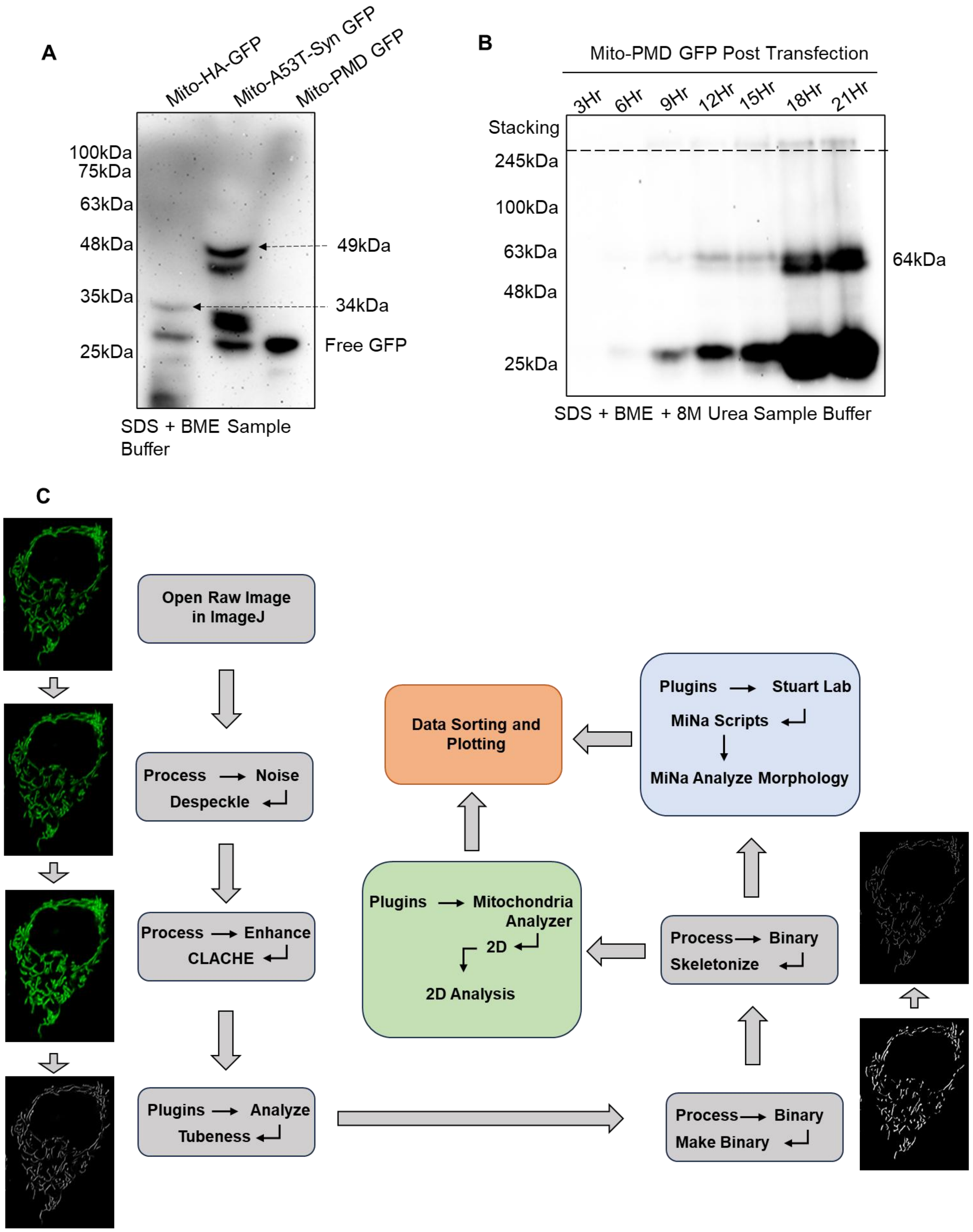
A
Mito-HA-GFP
Mito-A53T-Syn GFP
Mito-PMD GFP
100kDa
75kDa
63kDa
48kDa
35kDa
25kDa
49kDa
34kDa
Free GFP
SDS + BME Sample Buffer
B
Mito-PMD GFP Post Transfection
3Hr
6Hr
9Hr
12Hr
15Hr
18Hr
21Hr
Stacking
245kDa
100kDa
63kDa
48kDa
25kDa
64kDa
SDS + BME + 8M Urea Sample Buffer
C
Open Raw Image in ImageJ
Process → Noise
Despeckle
Process → Enhance
CLACHE
Plugins → Analyze
Tubeness
Process → Binary
Make Binary
Process → Binary
Skeletonize
Plugins → Mitochondria Analyzer
2D
2D Analysis
Plugins → Stuart Lab
MiNa Scripts
MiNa Analyze Morphology
Data Sorting and Plotting

Figure S3

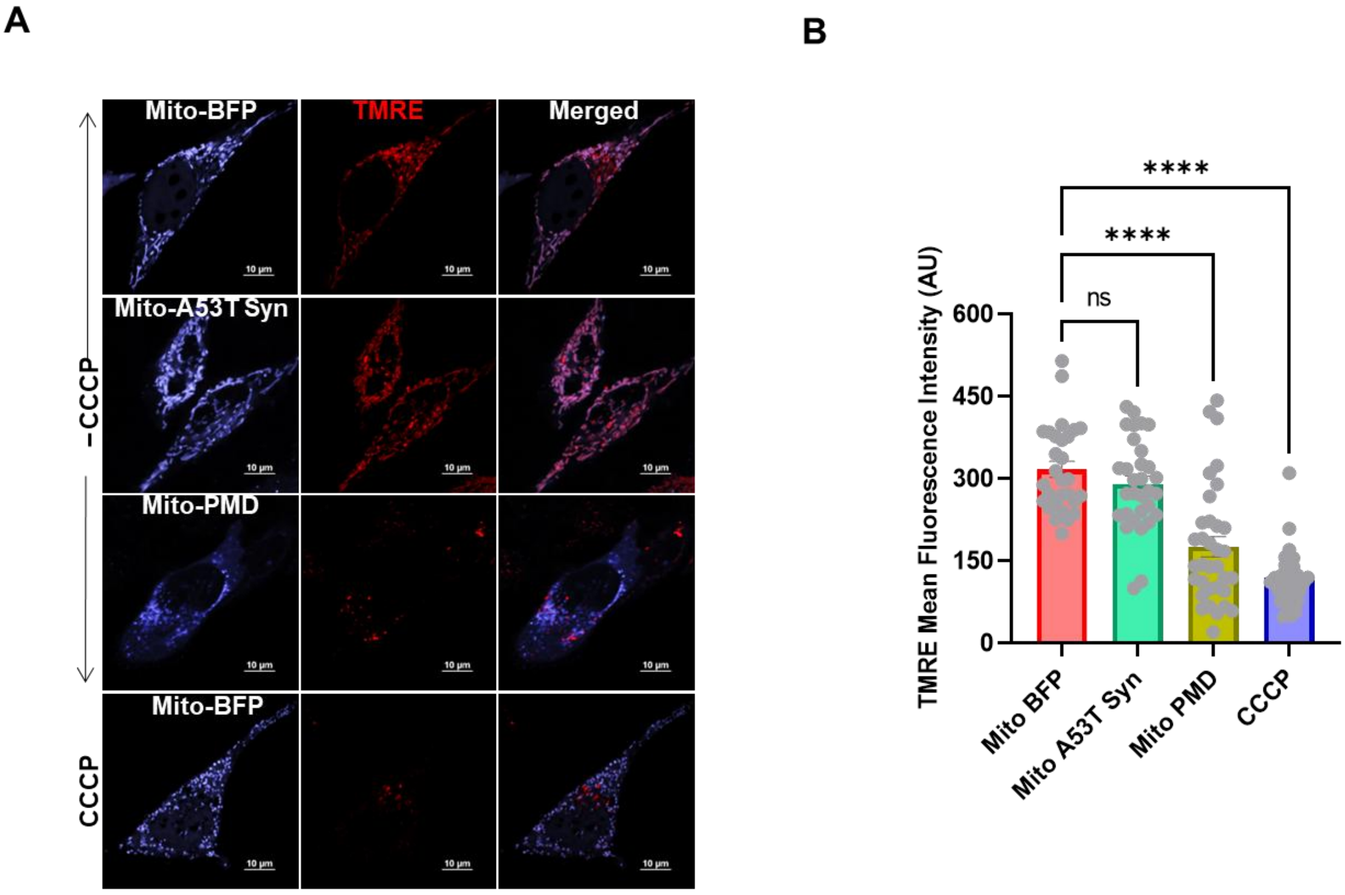
A
Mito-BFP
TMRE
Merged
Mito-A53T Syn
Mito-PMD
Mito-BFP
-CCCP
CCCP
10 μm
B
****
****
ns
TMRE Mean Fluorescence Intensity (AU)
600
450
300
150
0
Mito BFP
Mito A53T Syn
Mito PMD
CCCP

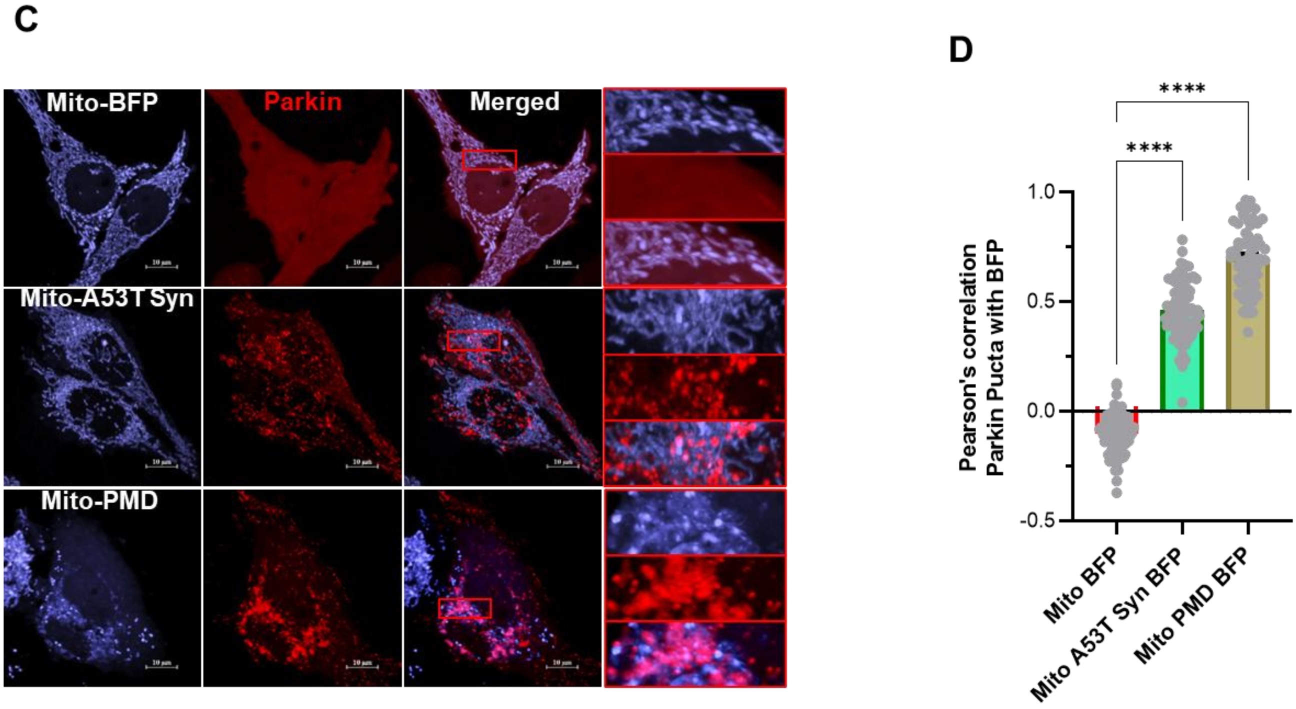
C
Mito-BFP
Parkin
Merged
Mito-A53T Syn
Mito-PMD
D
****
****
Pearson's correlation
Parkin Pucta with BFP
1.0
0.5
0.0
-0.5
Mito BFP
Mito A53T Syn BFP
Mito PMD BFP

Figure S4

A

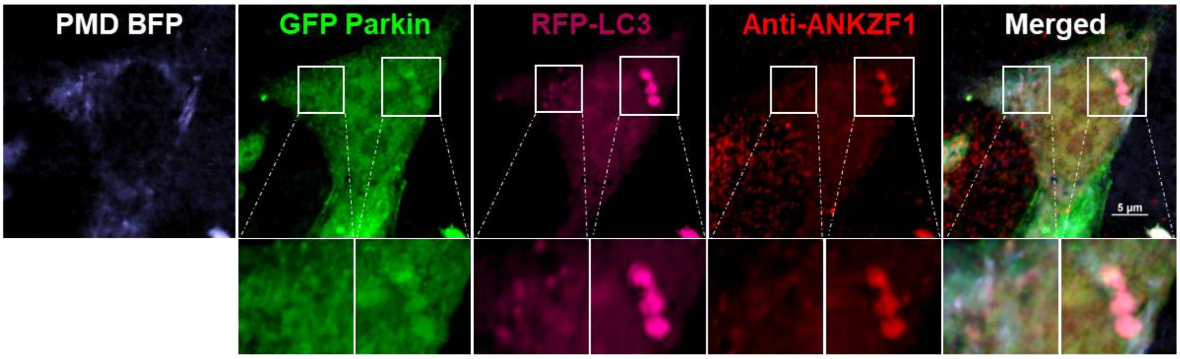

B

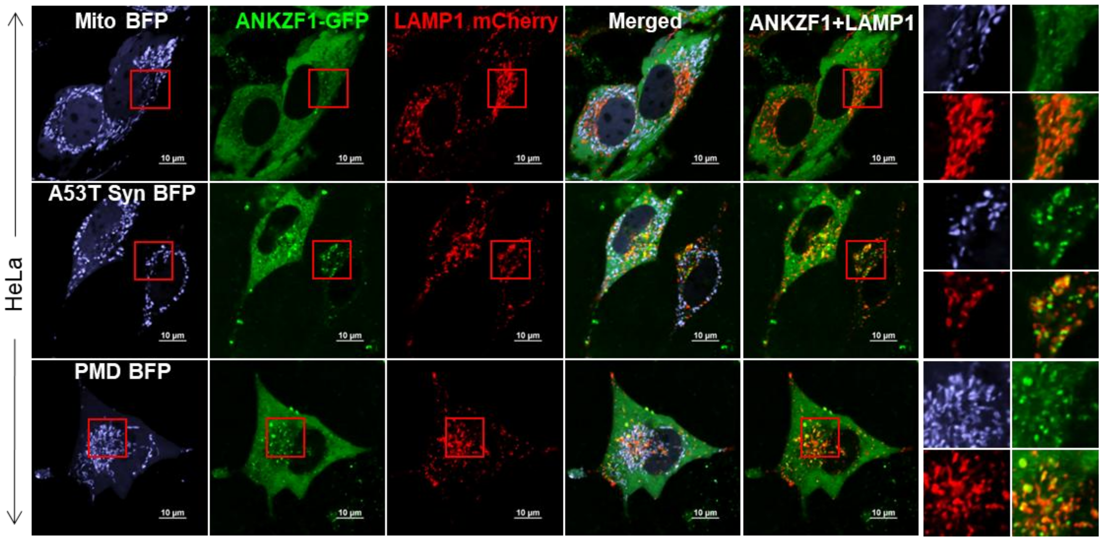

C

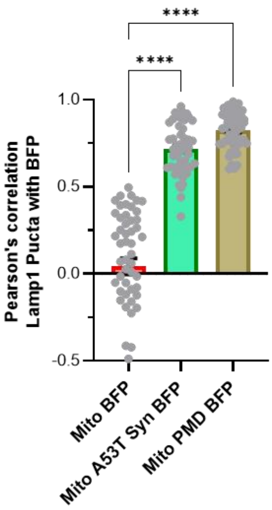

Figure S5

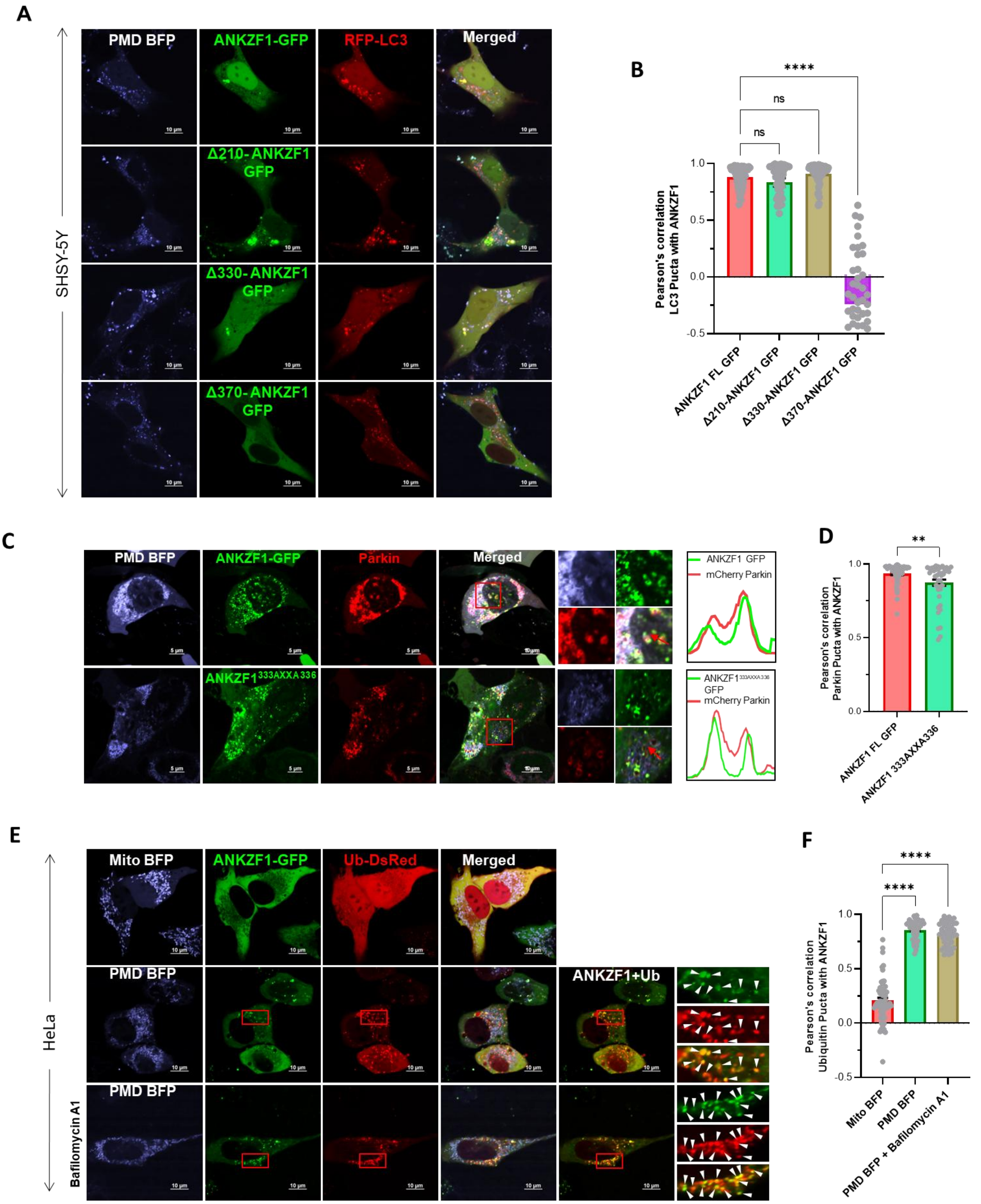

A
PMD BFP
ANKZF1-GFP
RFP-LC3
Merged
Δ210-ANKZF1 GFP
Δ330-ANKZF1 GFP
Δ370-ANKZF1 GFP
SHSY-5Y
10 μm
B
****
ns
ns
Pearson's correlation LC3 Pucta with ANKZF1
1.0
0.5
0.0
-0.5
ANKZF1 FL GFP
Δ210-ANKZF1 GFP
Δ330-ANKZF1 GFP
Δ370-ANKZF1 GFP
C
PMD BFP
ANKZF1-GFP
Parkin
Merged
ANKZF1333AXXA336
5 μm
ANKZF1 GFP
mCherry Parkin
ANKZF1333AXXA336 GFP
mCherry Parkin
D
**
Pearson's correlation Parkin Pucta with ANKZF1
1.0
0.5
0.0
ANKZF1 FL GFP
ANKZF1 333AXXA336
E
Mito BFP
ANKZF1-GFP
Ub-DsRed
Merged
PMD BFP
ANKZF1+Ub
HeLa
Bafilomycin A1
10 μm
F
****
****
Pearson's correlation Ubiquitin Pucta with ANKZF1
1.0
0.5
0.0
-0.5
Mito BFP
PMD BFP
PMD BFP + Bafilomycin A1

Figure S6

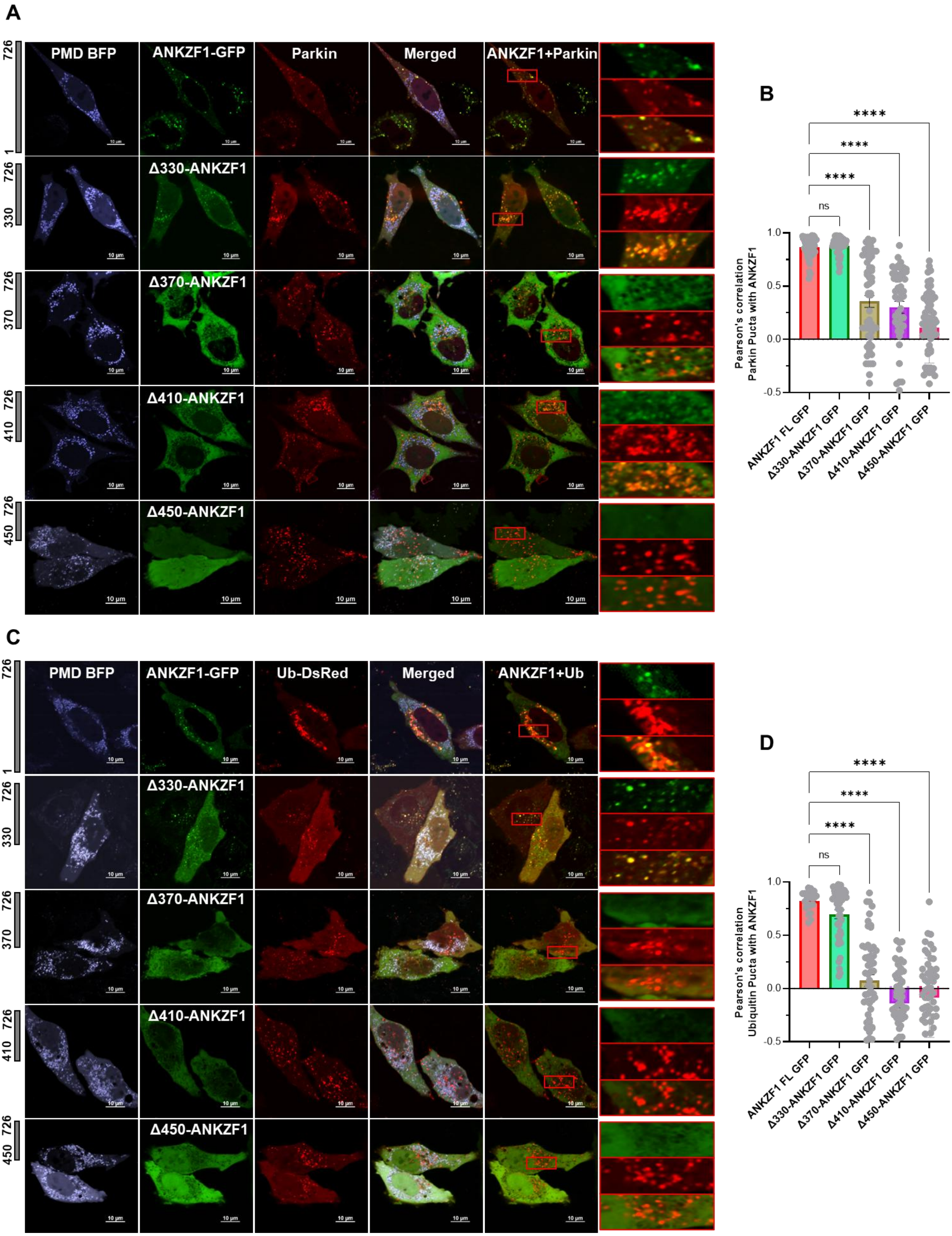
A
B
C
D
PMD BFP
ANKZF1-GFP
Parkin
Merged
ANKZF1+Parkin
Δ330-ANKZF1
Δ370-ANKZF1
Δ410-ANKZF1
Δ450-ANKZF1
Ub-DsRed
ANKZF1+Ub
Pearson's correlation Parkin Pucta with ANKZF1
Pearson's correlation Ubiquitin Pucta with ANKZF1
ns
****
ANKZF1 FL GFP
Δ330-ANKZF1 GFP
Δ370-ANKZF1 GFP
Δ410-ANKZF1 GFP
Δ450-ANKZF1 GFP

Figure S7

A

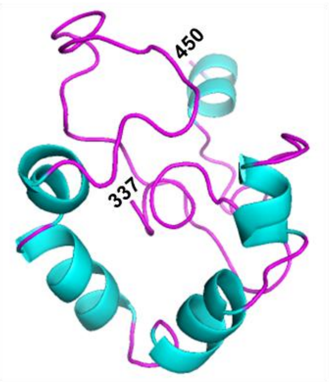

B

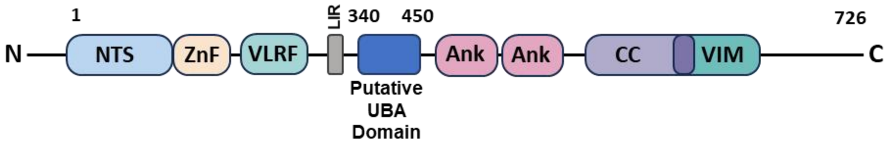

C

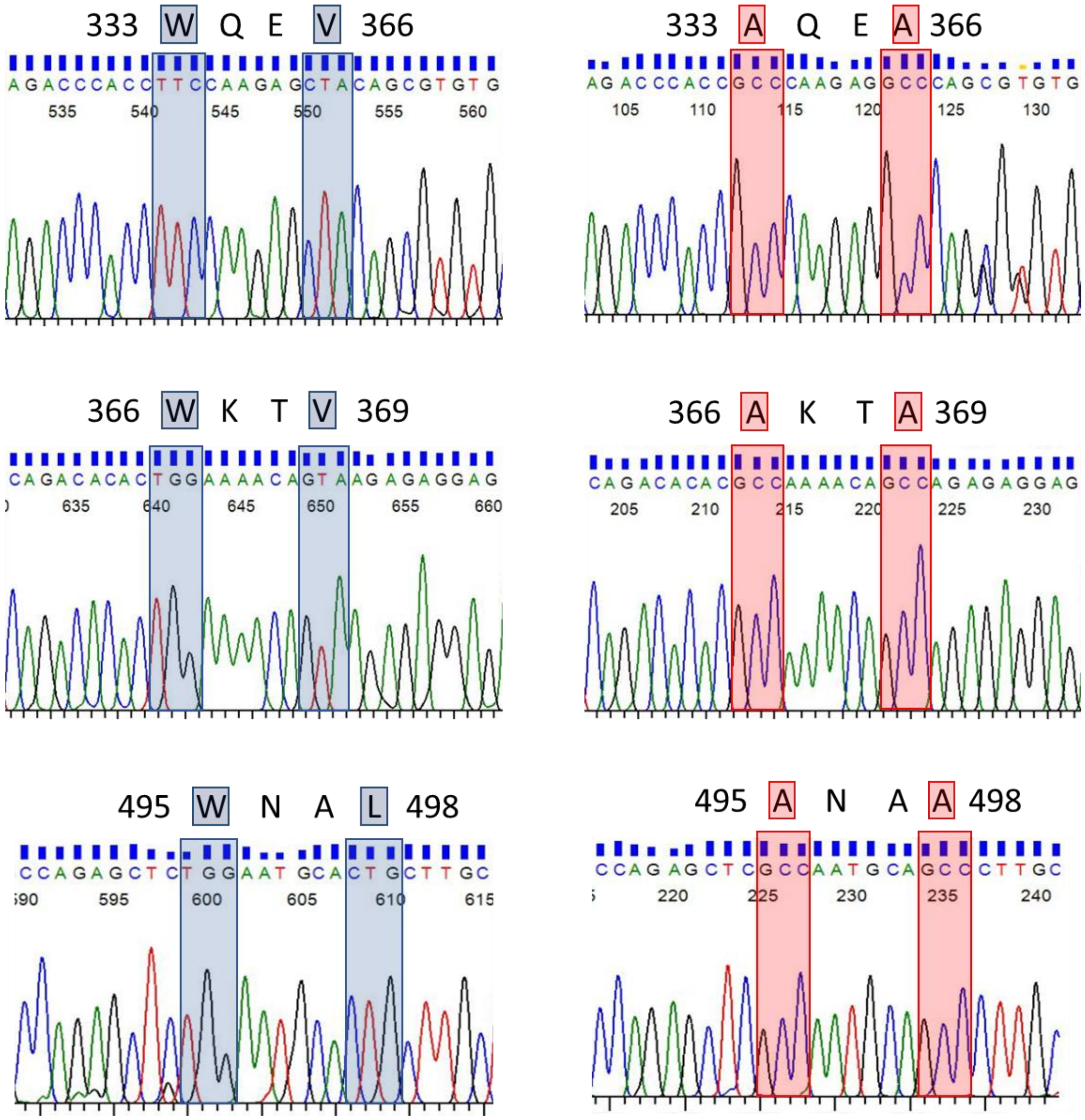

Figure S8

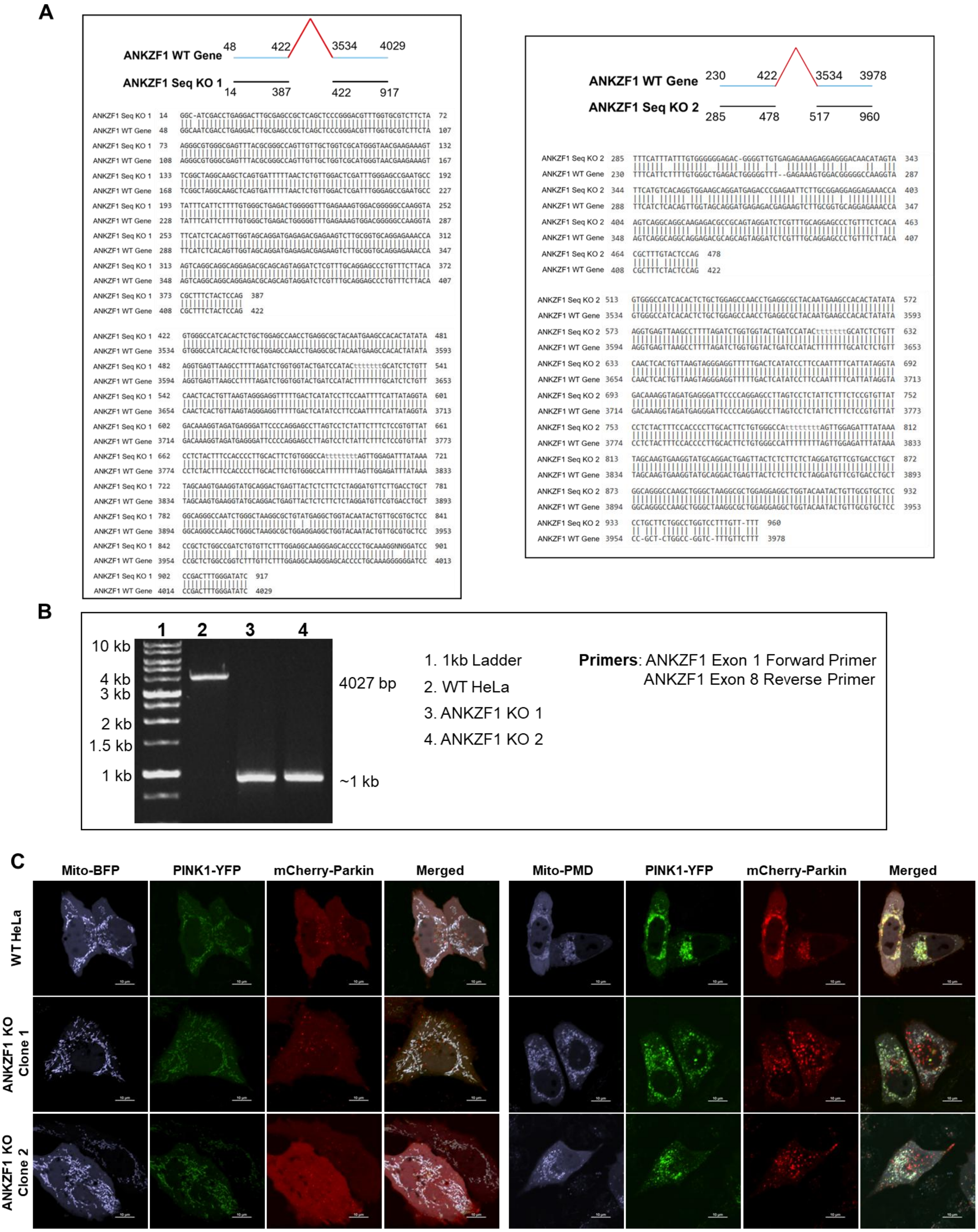
A
ANKZF1 WT Gene
48
422
3534
4029
ANKZF1 Seq KO 1
14
387
422
917
ANKZF1 WT Gene
230
422
3534
3978
ANKZF1 Seq KO 2
285
478
517
960
B
10 kb
4 kb
3 kb
2 kb
1.5 kb
1 kb
1
2
3
4
4027 bp
~1 kb
1. 1kb Ladder
2. WT HeLa
3. ANKZF1 KO 1
4. ANKZF1 KO 2
Primers: ANKZF1 Exon 1 Forward Primer
ANKZF1 Exon 8 Reverse Primer
C
Mito-BFP
PINK1-YFP
mCherry-Parkin
Merged
Mito-PMD
PINK1-YFP
mCherry-Parkin
Merged
WT HeLa
ANKZF1 KO Clone 1
ANKZF1 KO Clone 2

Figure S9

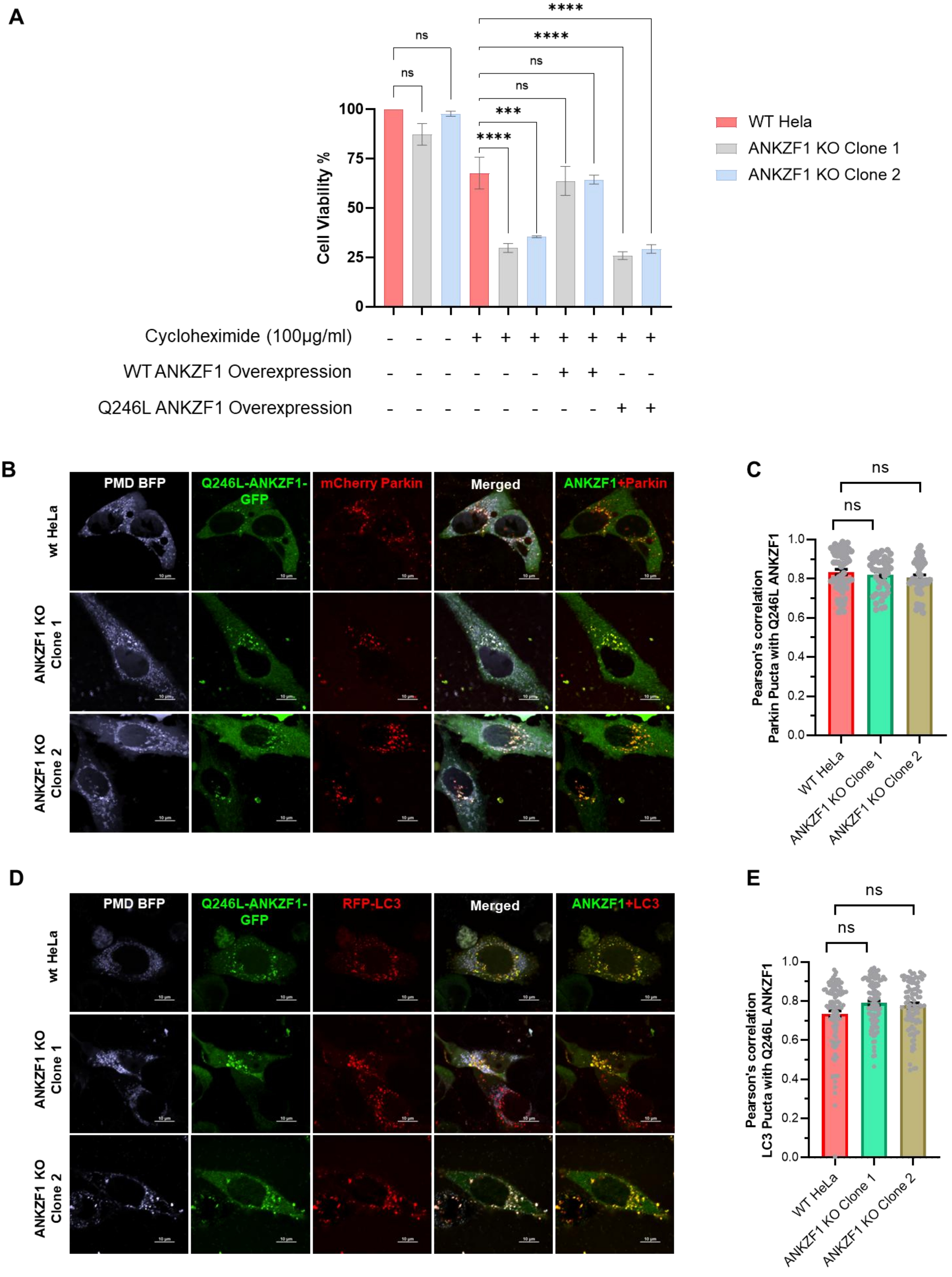

A
B
C
D
E
Cell Viability %
WT Hela
ANKZF1 KO Clone 1
ANKZF1 KO Clone 2
Cycloheximide (100µg/ml)
WT ANKZF1 Overexpression
Q246L ANKZF1 Overexpression
ns
****
***
PMD BFP
Q246L-ANKZF1-GFP
mCherry Parkin
Merged
ANKZF1+Parkin
wt HeLa
ANKZF1 KO Clone 1
ANKZF1 KO Clone 2
Pearson's correlation Parkin Pucta with Q246L ANKZF1
WT HeLa
RFP-LC3
ANKZF1+LC3
Pearson's correlation LC3 Pucta with Q246L ANKZF1

Figure S10

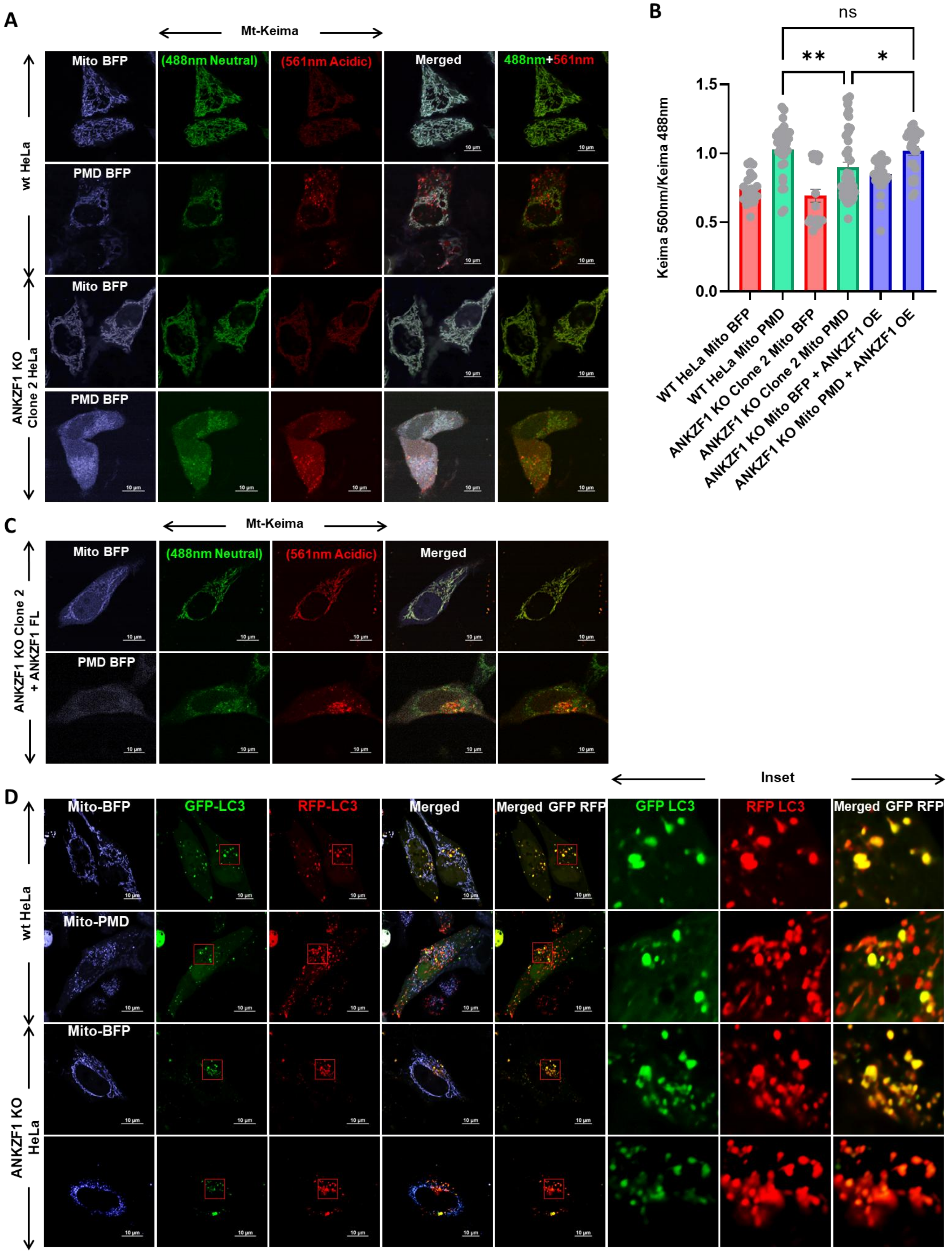
A
Mt-Keima
Mito BFP
(488nm Neutral)
(561nm Acidic)
Merged
488nm+561nm
PMD BFP
wt HeLa
ANKZF1 KO Clone 2 HeLa
B
ns
**
*
1.5
1.0
0.5
0.0
Keima 560nm/Keima 488nm
WT HeLa Mito BFP
WT HeLa Mito PMD
ANKZF1 KO Clone 2 Mito BFP
ANKZF1 KO Clone 2 Mito PMD
ANKZF1 KO Mito BFP + ANKZF1 OE
ANKZF1 KO Mito PMD + ANKZF1 OE
C
ANKZF1 KO Clone 2 + ANKZF1 FL
D
Inset
Mito-BFP
GFP-LC3
RFP-LC3
Merged
Merged GFP RFP
GFP LC3
RFP LC3
Mito-PMD
ANKZF1 KO HeLa

Figure S11

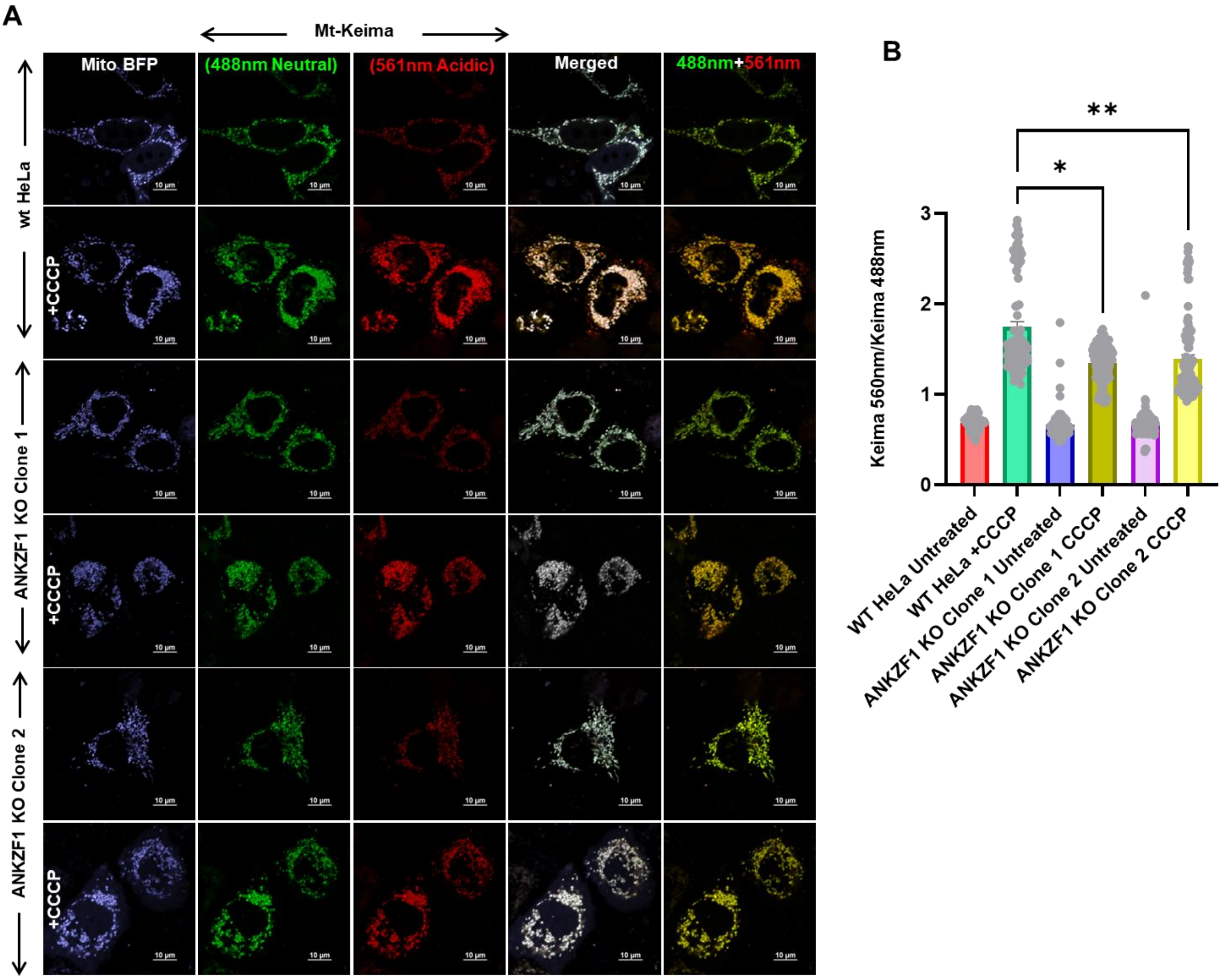
A
Mt-Keima
Mito BFP
(488nm Neutral)
(561nm Acidic)
Merged
488nm+561nm
wt HeLa
+CCCP
ANKZF1 KO Clone 1
+CCCP
ANKZF1 KO Clone 2
+CCCP
10 µm
B
Keima 560nm/Keima 488nm
0
1
2
3
*
**
WT HeLa Untreated
WT HeLa +CCCP
ANKZF1 KO Clone 1 Untreated
ANKZF1 KO Clone 1 CCCP
ANKZF1 KO Clone 2 Untreated
ANKZF1 KO Clone 2 CCCP

Supplementary figure legends

Figure S1

A. Images of HeLa cells obtained by confocal microscopy after transfection with ANKZF1-GFP and mCherry-Parkin and followed by treatment with H_2O_2, rotenone, sodium-azide, and paraquat at concentrations and conditions as mentioned in the materials and methods. In case of rotenone, sodium-azide, and paraquat treatments, cells show some scattered ANKZF1 and Parkin puncta formation, which partially co-localizes to mitochondrial network. **B.** TMRE-uptake based measurement of mitochondrial membrane potential of HeLa cells after treatment with CCCP, rapamycin, H_2O_2, rotenone, paraquat, and sodium-azide. Images show reduced TMRE uptake in comparison to control condition in all the treated conditions except H_2O_2 treatment. **C.** TMRE uptake of panel **(B)** was calculated by measuring the TMRE intensity and plotted as bar plots. CCCP showed the lowest TMRE uptake while H_2O_2 did not show any significant difference from the control cells and the rest of the treatments also showed reduced TMRE uptake. Values represent means ± SEM of N=3, data did not follow normal distribution, Kruskal-Wallis a non-parametric test with Dunn's multiple comparison test was performed to determine the mean differences, $^{**}P < 0.01$, $^{****}P < 0.0001$.

Figure S2

A. Western blot showing the presence of Mito-HA-GFP band at ~35kDa in the first lane and mito A53T α-synuclein-GFP band at ~48 kDa in the second lane, but in the third Mito-PMD-GFP band was not detected with anti-GFP antibody, all the samples were prepared in Laemmli buffer. **B.** HeLa cells were transfected with Mito-PMD-GFP, and cells were collected at every three hours intervals of transfection till 21 hours; cells were lysed in 2x Laemmli buffer supplemented with 8M Urea. Western blot shows the presence of GFP-tagged Mito-PMD protein at ~64 kDa. **C.** Schematic workflow of mitochondrial mean branch length measurement using ImageJ.

Figure S3

A. Left panel in all four rows shows the expression of BFP tagged control and stressor proteins (Mito-BFP, Mito-A53T-α-synuclein, and Mito-PMD). Second panel from left, shows the TMRE intensity, cells showing TMRE staining contain healthy mitochondria with intact membrane potential. CCCP-treated cells were used as a negative control for TMRE staining. Expression of Mito-PMD shows significant loss of mitochondrial membrane potential. However, Mito-A53T-α-synuclein expression does not cause much change in the mitochondrial membrane potential. **B.** TMRE uptake shown in panel **(A)** was calculated by measuring the TMRE intensity and plotted. CCCP-treated and PMD-expressing cells show lowest TMRE intensity, Mito-A53T-α-synuclein expression does not cause any significant change in TMRE intensity. Values represent means ± SEM of N >3, data did not follow normal distribution, Kruskal-Wallis a non-parametric test with Dunn's multiple comparison test was performed to determine the mean differences, $^{**}P < 0.01$, $^{****}P < 0.0001$. **C.** mCherry-Parkin was co-expressed in HeLa cells expressing either control protein (Mito-BFP), or stressor proteins (A53T-α-synuclein-BFP, or PMD-BFP). In the control panel (Mito-BFP), mCherry-Parkin shows diffused cytosolic expression, but in stressed conditions, lower two panels (A53T α-synuclein BFP and PMD BFP) show a high level of mCherry parkin puncta recruitment on mitochondria indicating increased mitophagic flux due to proteotoxic stress. Inset shows zoomed in boxed regions of the merged panel. **D**. Pearson's correlation co-efficient profile of co-localization between mCherry-Parkin and mito-BFP shows significant Parkin recruitment on mitochondria during A53T-Synuclein and PMD stress conditions. Values represent means ± SEM of N>3, dataset was following a normal distribution, One-way ANOVA with Bonferroni's multiple comparison test was performed to determine the mean differences, $^{****}P < 0.0001$.

Figure S4

A. Cells were transfected with Mito PMD BFP, GFP Parkin and RFP-LC3, followed by the ANKZF1 probing by Anti-ANKZF1 antibody, result shows various co-localized events of Parkin, LC3, and ANKZF1 during mitochondrial proteotoxic stress condition. **B.** HeLa cells were co-transfected with LAMP1 mCherry and ANKZF1 GFP along with mitochondrial

stressors protein, top panel shows control condition, ANKZF1 remains in cytosol and no co-localization between ANKZF1 and LAMP1 were observed. Middle and lower panel shows A53T Syn and PMD stressor conditions, in both results ANKZF1 formed puncta and shows significant co-localized events with LAMP1-mCherry. **C**. Pearson's correlation co-efficient profile of co-localization between LAMP1-mCherry and ANKZF1-GFP shows significant co-localization during A53T-Synuclein and PMD-induced proteotoxic stress condition. Values represent means ± SEM of N>3. Dataset was following a normal distribution. One-way ANOVA with Bonferroni's multiple comparison test was performed to determine the mean differences, ****P < 0.0001.

Figure S5

A. ANKZF1 truncation mutants (WT-ANKZF1, Δ210-ANKZF1, Δ330-ANKZF1, and Δ370-ANKZF1) were co-expressed with RFP-LC3 in SHSY-5Y cells in the presence of PMD-induced proteotoxic stress. Stressed cells show ANKZF1 puncta formation by all the truncation mutants except Δ370-ANKZF1. **B**. Pearson's correlation co-efficient profile of co-localization between RFP-LC3 and ANKZF1-GFP shows Δ370-ANKZF1 fails to localize with LC3. Values represent means ± SEM of N>3, dataset did not follow a normal distribution, Kruskal-Wallis a non-parametric test with Dunn's multiple comparison test was performed to determine the mean differences, ns: Non significance, ****P < 0.0001. **C.** WT ANKZF1 and F333A-L336A mutant of ANKZF1 were co-expressed with mCherry Parkin and Mito-PMD, showing co-localization (line profile) ANKZF1 and Parkin in both the conditions. **D**. Pearson's correlation co-efficient profile of co-localization between mCherry-Parkin and WT-ANKZF1-GFP and LIR mutant ANKZF1-GFP. Values represent means ± SEM of N>3, unpaired students t-test was used to analyze the mean difference, **P < 0.01. **E.** ANKZF1 FL and Ub-DsRed were co-expressed in HeLa cells along with PMD and control protein; the top panel shows Mito-BFP control where ANKZF1 remains in the cytosol. The middle and lower panel shows PMD and bafilomycin A1 treated PMD conditions, showing ANKZF1 puncta formation and co-localization of ANKZF1 puncta with Ub-DsRed puncta, suggesting ANKZF1 and

ubiquitin interaction. **F**. Pearson's correlation co-efficient profile of co-localization between Ubiquitin-DsRed and ANKZF1-GFP shows significant co-localization during PMD stress condition. Values represent means ± SEM of N>3, dataset did not follow a normal distribution, Kruskal-Wallis a non-parametric test with Dunn's multiple comparison test was performed to determine the mean differences, ns: Non significance, ****P < 0.0001.

Figure S6

A. ANKZF1 recruitment and interaction with Parkin during PMD-induced mitochondrial proteotoxic stress were checked after expressing the N-terminal truncation mutants of ANKZF1. ANKZF1-FL shows puncta formation that co-localizes with mCherry-Parkin puncta during proteotoxic stress (panel A, upper row). Δ330-ANKZF1 truncation mutants also show similar behaviour and significantly co-localize with Parkin puncta (panel A, second row from the top) like the full-length protein. Δ370-ANKZF1, Δ410-ANKZF1, and Δ450-ANKZF1 show a diffused cytosolic pattern indicating no interaction with Parkin. **B**. Pearson's correlation co-efficient profile of co-localization between mCherry-Parkin and ANKZF1-GFP show significant loss in the interaction between ANKZF1 and Parkin when at least initial 370 amino acids were deleted. Values represent means ± SEM of N>3, dataset did not follow a normal distribution, Kruskal-Wallis a non-parametric test with Dunn's multiple comparison test was performed to determine the mean differences, ns: Non significance, ****P < 0.0001. **C**. ANKZF1 recruitment and interaction with Ubiquitin during PMD-induced mitochondrial proteotoxic stress were checked after expressing the truncation mutants of ANKZF1. ANKZF1-FL shows puncta formation that co-localize with Ub-DsRed puncta during proteotoxic stress (panel C, upper row). Δ330-ANKZF1 truncation mutants also show similar behavior and significantly co-localize with Parkin puncta (panel C, second rows from the top) like the full-length protein. Δ370-ANKZF1, Δ410-ANKZF1, and Δ450-ANKZF1 shows a diffused cytosolic pattern indicating no interaction with

ubiquitin. **D**. Pearson's correlation co-efficient profile of co-localization between Ubiquitin-DsRed and ANKZF1-GFP shows significant loss in the interaction between ANKZF1 and ubiquitin in Δ370-ANKZF1 overexpression. Values represent means ± SEM of N>3, dataset did not follow a normal distribution, Kruskal-Wallis a non-parametric test with Dunn's multiple comparison test was performed to determine the mean differences, ns: Non significance, ****P < 0.0001.

Figure S7.

A. I-TASSER predicted-modelled structure of 337-450 residues of ANKZF1, showing formation of α-helices. **B**. Schematic diagram of ANKZF1 domain structure showing the presence of putative UBA domain **C**. Chromatogram showing sequencing confirmation of ANKZF1 LIR mutants generated by site-directed mutagenesis. Left panel shows wild-type sequences with blue highlights at the LIR site, the right panel shows mutant sequences, and red highlights show the mutations being incorporated.

Figure S8

A. *ANKZF1* gene of both the KO clone cells were sequenced and aligned with the wild type ANKZF1 sequence, showing a gap of around 3 kb suggesting successful deletion of part of the genomic DNA. **B.** Agarose gel electrophoresis of PCR products amplified from genomic DNA of WT HeLa cells and *ANKZF1* KO cells. PCR was done by using ANKZF1 Exon 1 forward and Exon 8 reverse primers. In the WT HeLa cells, a ~4 kb band was observed, while in KO cells ~1 kb band was observed showing deletion of ~3 kb fragment. **C.** Left panel shows healthy mitochondrial control (Mito-BFP), and PINK1-YFP and mCherry Parkin expression was assessed in wild type and both the *ANKZF1* knockout clones. The right panel shows PINK1 and Parkin expression during mitochondrial proteotoxic stress due to expression of Mito-PMD. In the control panel, no significant change in the PINK1 or Parkin expression in

the KO cells is observed, while in the stressed condition, PINK1 and Parkin recruitment on the mitochondria was significantly increased in all three conditions; no overall cell-to-cell variability was observed.

Figure S9.

A. Bar plot showing the cell viability percentage of wt HeLa and *ANKZF1* KO clones, first three bars showing untreated control, bar 4-6 showing cells exposed with translation inhibitor cycloheximide (100µg/ml) for 24 hours, where *ANKZF1* KO cells showing sensitivity to cycloheximide treatment. Bar 7 and 8 shows *ANKZF1* KO clones were rescued with the overexpression of wild type ANKZF1 and treated with cycloheximide (100µg/ml) for 24 hours, showing overexpression of WT ANKZF1 helps in the cell survival. Last 2 bars represent the conditions where *ANKZF1* KO clones were rescued with the overexpression of tRNA hydrolase deficient mutant Q246L ANKZF1 and treated with cycloheximide (100µg/ml) for 24 hours, here the cell death was not rescued by overexpression of mutant ANKZF1. Values represent means ± SEM of N =3, data was following a normal distribution, one-way ANOVA with Bonferroni correction for multiple comparison was performed to determine the mean differences, ns: non-significant differences, ***P < 0.001, ****P < 0.0001. **B.** Q246L ANKZF1 was overexpressed and checked its interaction with Parkin, during mitochondrial proteotoxic stress Mito-PMD in wt HeLa and *ANKZF1* Knockout clones, in every condition, Q246L ANKZF1-GFP was able to co-localize with mCherry-Parkin. **C.** Pearson's correlation co-efficient profile of Q246L -ANKZF1-GFP with mCherry-Parkin in wt HeLa and both the *ANKZF1* KO clones showing no significant changes. **D.** Q246L-ANKZF1 was overexpressed and checked its interaction with RFP-LC3, during mitochondrial proteotoxic stress Mito-PMD in wt HeLa and *ANKZF1* Knockout clones, in every condition Q246L ANKZF1 GFP was able to co-localize with RFP-LC3. **E.** Pearson's correlation co-efficient profile of Q246L ANKZF1 GFP with RFP-LC3 in wt HeLa and both the *ANKZF1* KO clones showing no significant changes. Values represent means ± SEM of N >3, data did not follow a normal distribution, Kruskal-

Wallis a non-parametric test with Dunn's multiple comparison test was performed to determine the mean differences, ns: non-significant differences.

Figure S10

A. The mitophagy rate of WT HeLa cells and *ANKZF1* KO cells were compared by using pH dependent Mt-Keima protein as explained in the results. KO clone 2 cells showed functional mitophagy events but relatively lowered mitophagy events in comparison to WT HeLa cells when PMD protein was expressed to induce mitochondrial proteotoxic stress. **B.** Keima560/Keima488 Ratiometric analysis suggests PMD expressed WT-Hela cells showing significantly higher 560/488 ratio in comparison to the KO cells corresponding high mitophagy rate. **C.** ANKZF1 was overexpressed in ANKZF1 KO cells and Mt-Keima based mitophagy turnover was analysed, in PMD expressed cells, Keima 561 signal behaving like WT HeLa cells, suggesting reversal of phenotype in presence of ANKZF1. Values represent means ± SEM of N >3, data did not follow a normal distribution, Kruskal Wallis a non-parametric test with Dunn's multiple comparison test was performed to determine the mean differences, $*P < 0.05$, $**P < 0.01$. **D.** WT HeLa cells and ANKZF1 KO cells were transfected with tf-LC3 (GFP and RFP tagged LC3) along with control and PMD-BFP overexpression in mitochondria. In both the cell lines (WT and KO) the overall autophagy flux is not compromised in the presence of PMD proteotoxic stress, almost same number of red LC3 puncta were observed suggesting functional autophagy.

Figure S11.

A and B. Mt-Keima was expressed in WT HeLa cells and ANKZF1 KO cells (clone 1 and clone 2) followed by the CCCP treatment to induce mitophagy, followed by the confocal microscopy. Results show in WT cells Keima488 to Keima560 shift is higher than the KO cells, suggesting higher mitophagy rate in WT cells in comparison to KO cells. Values represent means ± SEM of N >3, data were not following normal distribution, Kruskal Wallis a non-parametric test with

Dunn's multiple comparison test was performed to determine the mean differences, *P < 0.05, **P < 0.01.

Supplementary table 1.

List of plasmid constructs used in this work

Construct Name	Vector Backbone	Source	Addgene Number
EBFP2-Mito-7	EBFP2	Addgene	55248
mCherry-Parkin	mCherry-C1	Addgene	23956
Mito HA-GFP	pcDNA3.1	Addgene	67486
mCherry-Mito-7	mCherry	Addgene	55102
ptf-LC3	EGFP-C1	Addgene	21074
mt-mKeima	pHAGE	Addgene	131626
RFP-LC3		Received as a gift from Professor Oishee Chakrabarti's lab, SINP, Kolkata	
Mito-A53T-Synuclein-GFP	EGFPN1	This study	
Mito-PMD-GFP	EGFPN1	This study	
Mito-A53T-Synuclein-BFP	EBFP	This study	
Mito-PMD-BFP	EBFP	This study	
ANKZF1-FL-GFP	pcDNA3.1	This study	
Δ210-ANKZF1-GFP	EGFPN1	This study	
Δ330-ANKZF1-GFP	EGFPN1	This study	
Δ370-ANKZF1-GFP	EGFPN1	This study	
Δ410-ANKZF1-GFP	EGFPN1	This study	
Δ450-ANKZF1-GFP	EGFPN1	This study	
ANKZF1-333AXXA336-GFP	EGFPN1	This study	
ANKZF1-366AXXA369-GFP	EGFPN1	This study	
ANKZF1-495AXXA498-GFP	EGFPN1	This study	
Q246L ANKZF1-GFP	EGFPN1	This study	
Ub G76V GFP	EGFPN1	Addgene	11941
Ub G76V DsRed	DsRedN1	This Study	
LAMP1-emiRFP670	emiRFP670	Addgene	136570
LAMP1-mCherry	mCherryN1	This Study	
EGFP-Parkin	EGFPC1	This Study	

Supplementary table 2.

List of reagents and antibody used in this paper.

Reagents and Antibody	Made	Cat number
Lipofectamine™ 2000	Thermo	11668027
H_2O_2	MP Bio	Addgene
Rotenone	Sigma-Aldrich	R8875
Rapamycin	Sigma-Aldrich	553210
CCCP	Sigma-Aldrich	C2759
Paraquat	Sigma-Aldrich	856177
Na-Azide	Himedia	MB075
Cycloheximide	MP Bio	194527
Thiazolyl blue tetrazolium bromide (MTT)	Sigma	M2128
MitoTracker Far Red	Invitrogen	M22426
TMRE	Invitrogen	T669
Anti-ANKZF1	Sigma	HPA035208
Anti-GFP	Generated in Lab	
Anti-LC3	Cell signalling technology	3868T
Anti-Parkin	Cell signalling technology	2132S
Anti-Actin	Elabscience	E-AB-40517
Alexa-647	Invitrogen	A21245

Supplementary table 3

Primer Name	Primer Sequence
ANKZF1 EcoRI Forward	5’CCCGAATTCATGTCGCCGGCTCCAGATGCAGCCCCGGCTCC3’
211 ANKZF1 EcoRI Forward	5’ CCCGAATTCATGGCTGCAGCTGGGCACTTTGCTGGTG 3’
330-ANKZF1 XhoI Forward	5’ GTTCTCGAGATGCCCACCTTCCAAGAGCTAC 3’
370-ANKZF1 XhoI Forward	5’ GTTCTCGAGATGGAGGAGAGAAAGAAGCCTAC 3’
410-ANKZF1 XhoI Forward	5’ GTTCTCGAGATGTTTAGGTAGAGTTGGAGC 3’
450-ANKZF1 XhoI Forward	5’ GTTCTCGAGATGGAGGCTGGGGCACATCGG3 3’
ANKZF1 No stop KpnI Reverse	5’ CCCGGTACCCGGGAAGAGGGCCTCCCTGCCTGACGGCGATG 3’

ANKZF1 F333A and L336A SDM Forward	5’ AGACCCACCGCCCAAGAGGCCCAGCGTGTG 3’
ANKZF1 F333A and L336A SDM Reverse	5’ CACACGCTGGGCCTCTTGGGCGGTGGGTCT 3’
ANKZF1 W366A and V369A SDM Forward	5’ CAGACACACGCCAAAACAGCCAGAGAGGAG 3’
ANKZF1 W366A and V369A SDM Reverse	5’ CTCCTCTCTGGCTGTTTTGGCGTGTGTCTG 3’
ANKZF1 W495A and L498A SDM Forward	5’ CCAGAGCTCGCCAATGCAGCCCTTGCTGCT 3’
ANKZF1 W495A and L498A SDM Reverse	5’ AGCAGCAAGGGCTGCATTGGCGAGCTCTGG 3’
ANKZF1 Q246L Forward	5’ GGGCACAGCCCTGGGGCTTCGGG 3’
ANKZF1 Q246L Reverse	5’ CCCGAAGCCCCAGGGCTGTGCCC 3’
ANKZF1 Exon 1 Forward	5’ CGCTGCTCCGTAGTGACGG 3’
ANKZF1 Exon 8 Reverse	5’ CCCGGTACCCGGATATCCCAAAGTCGGGGATCC 3’
ANKZF1 sgRNA 1 Forward Oligo	5’ CACCGCTTGGCCCGAACCGTATAG 3’
ANKZF1 sgRNA 1 Reverse Oligo	5’ AAACCTATACGGTTCGGGCCAAGC 3’
ANKZF1 sgRNA 2 Forward Oligo	5’ CACCGAGTGTGATGGCCCACCTC 3’
ANKZF1 sgRNA 2 Reverse Oligo	5’ AAACGAGGTGGGCCATCACACTC 3’
ANKZF1 sgRNA 3 Forward Oligo	5’ CACCGTTTCTGCTCAGCCATGTCGC 3’
ANKZF1 sgRNA 3 Reverse Oligo	5’ AAACGCGACATGGCTGAGCAGAAAC 3’
ANKZF1 sgRNA 4 Forward Oligo	5’ CACCGTCAAACAGGGAGATCGACGC 3’
ANKZF1 sgRNA 4 Reverse Oligo	5’ AAACGCGTCGATCTCCCTGTTTGAC 3’
